# Rapid, visual, label-based biosensor platform for identification of hepatitis C virus in clinical applications

**DOI:** 10.1186/s12866-024-03220-9

**Published:** 2024-02-28

**Authors:** Yuanfang Shi, Qingxue Zhou, Shilei Dong, Qi Zhao, Xue Wu, Peng Yang, Xiaoyan Zeng, Xinggui Yang, Yan Tan, Xinhua Luo, Zhenghua Xiao, Xu Chen

**Affiliations:** 1 The Second Clinical Medical College, Guizhou University of Traditional Chinese Medicine, Guiyang, Guizhou, 550003 People’s Republic of China; 2 Central Laboratory of the Second Affiliated Hospital, Guizhou University of Traditional Chinese Medicine, Guiyang, Guizhou, 550003 People’s Republic of China; 3 Clinical Laboratory, Hangzhou Women’s Hospital, Hangzhou, Zhejiang 310008 People’s Republic of China; 4 Department of Clinical Laboratory, Zhejiang Hospital, Hangzhou, Zhejiang 310013 People’s Republic of China; 5 Department of gastroenterology, the Second Affiliated Hospital, Guizhou University of Traditional Chinese Medicine, Guiyang, Guizhou, 550003 People’s Republic of China; 6 Department of Scientific Research, the Second Affiliated Hospital, Guizhou University of Traditional Chinese Medicine, Guiyang, Guizhou, 550003 People’s Republic of China; 7 Clinical Laboratory, the Second Affiliated Hospital, Guizhou University of Traditional Chinese Medicine, Guiyang, Guizhou, 550003 People’s Republic of China; 8 Experiment Center, Guizhou Provincial Centre for Disease Control and Prevention, Guiyang, Guizhou, 550004 People’s Republic of China; 9 Clinical Laboratory, Guizhou Provincial Center for Clinical Laboratory, Guiyang, Guizhou, 550002 People’s Republic of China; 10 Department of Infectious Disease, Guizhou Provincial People’s Hospital, Guiyang, Guizhou, 550002 People’s Republic of China

**Keywords:** Hepatitis C virus, Reverse transcription loop-mediated isothermal amplification, Biosensor, Point-of-care platform, Limit of detection

## Abstract

**Objectives:**

In the current study, for the first time, we reported a novel HCV molecular diagnostic approach termed reverse transcription loop-mediated isothermal amplification integrated with a gold nanoparticles-based lateral flow biosensor (RT-LAMP-AuNPs-LFB), which we developed for rapid, sensitive, specific, simple, and visual identification of HCV.

**Methods:**

A set of LAMP primer was designed according to 5’untranslated region (5’UTR) gene from the major HCV genotypes 1b, 2a, 3b, 6a, and 3a, which are prevalent in China. The HCV-RT-LAMP-AuNPs-LFB assay conditions, including HCV-RT-LAMP reaction temperature and time were optimized. The sensitivity, specificity, and selectivity of our assay were evaluated in the current study. The feasibility of HCV-RT-LAMP-AuNPs-LFB was confirmed through clinical serum samples from patients with suspected HCV infections.

**Results:**

An unique set of HCV-RT-LAMP primers were successfully designed targeting on the 5’UTR gene. The optimal detection process, including crude nucleic acid extraction (approximately 5 min), RT-LAMP reaction (67℃, 30 min), and visual interpretation of AuNPs-LFB results (~ 2 min), could be performed within 40 min without specific instruments. The limit of detection was determined to be 20 copies per test. The HCV-RT-LAMP-AuNPs-LFB assay exhibited high specificity and anti-interference.

**Conclusions:**

These preliminary results confirmed that the HCV-RT-LAMP-AuNPs-LFB assay is a sensitive, specific, rapid, visual, and cost-saving assay for identification of HCV. This diagnostic approach has great potential value for point-of-care (POC) diagnostic of HCV, especially in resource-challenged regions.

**Supplementary Information:**

The online version contains supplementary material available at 10.1186/s12866-024-03220-9.

## Introduction

Hepatitis C caused by infection with hepatitis C virus (HCV) is a global public health problem [1]. Approximately 71 million individuals live with chronic HCV infection worldwide, with approximately 1.75 million new cases developing each year [2]. HCV transmission is most commonly associated with blood transfusions, direct percutaneous exposure to blood, injection drug use, and maternal-fetal transmission [3]. People infected with HCV often become chronic carriers and progress to severe liver diseases, such as hepatitis, liver cirrhosis, and hepatocellular carcinoma [2, 4]. New and effective treatment using oral direct-acting antivirals (DAAs) has paved the way to cure hepatitis C with few side effects [5]. Unfortunately, fewer than 20% of those living with HCV are aware they are infected. A current challenge is to engage and screen as many people as possible in need of treatment, especially in low and middle-income countries [6]. Development of a simple, rapid, specific, sensitive, and affordable point-of-care (POC) technique for screening of HCV is fundamental to achieve the World Health Organization (WHO) 2030 elimination targets.

Traditional laboratory-based diagnosis of HCV infection based on immunoassays relies of detection of HCV antibody (HCV-Ab) and HCV core antigen (HCV-cAg) [7]. However, the sensitivity is low and the development of HCV-Ab requires 30–60 days following exposure to HCV [7, 8]. Real-time polymerase chain reaction (RT-PCR) is the new diagnostic gold standard for confirmation of HCV infection, reflecting the technique’s higher sensitivity, specificity, and capability for automation [7]. HCV RNA appears in the bloodstream within 2–14 days following exposure to HCV [8]. Nevertheless, this assay is inapplicability in many resource-constrained settings limits their application in many resource-constrained areas, primarily because of the high costs of thermo-cycling instruments and need for trained technicians.

Loop-mediated isothermal amplification (LAMP) is an advanced nucleic acid isothermal amplification approach that has been implicated as an attractive alternative to traditional PCR-related techniques. LAMP is promising as the basis of POC testing due to its high specificity, sensitivity, ease of use, and less expensive cost [9, 10]. In addition, LAMP has been applied to identify several human important pathogens, such as severe acute respiratory syndrome coronavirus 2 (SARS-CoV-2), human immunodeficiency virus (HIV), and human influenza virus [11–13]. The gold nanoparticles-based lateral flow biosensor (AuNPs-LFB) is a paper-based platform that has demonstrated huge potential as an advanced POC testing tool. The AuNPs-LFB platform is portable, easy to construct and operate, highly sensitive and specific, capable of rapid detection, and provides a naked-eye visual readout [14, 15]. More importantly, the AuNPs-LFB platform can be applied for the identification of various biomarkers, such as antigens, antibodies, nucleic acids, and infectious pathogens [16, 17].

**Fig. 1:**
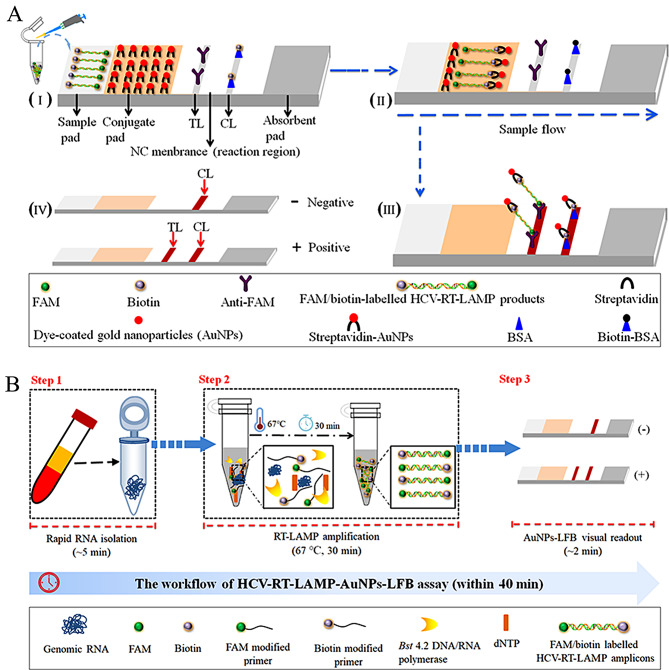
Schematic diagram of HCV-RT-LAMP-AuNPs-LFB assay’s principle. (**A**) Schematic diagram of AuNPs-LFB principles for the interpretation of HCV-RT-LAMP outcomes. I, HCV-RT-LAMP amplification products (2.0 µl) and running buffer (100 µl) are added simultaneously on the sample pad. II, Due to capillary action, the running buffer containing HCV-RT-LAMP products move forward onto the conjugate pad and nitrocellulose (NC) membrane. The dye streptavidin-coated gold nanoparticles (streptavidin-AuNPs) are rehydrated and integrate with FAM/biotin labeled HCV-RT-LAMP products. III, The FAM/biotin-labeled HCV-RT-LAMP products are captured by anti-FAM at the test line (TL). Streptavidin-AuNPs are captured by biotin-BSA at the control line (CL). IV, Interpretation of the HCV-RT-LAMP-AuNPs-LFB assay. HCV-positive results are indicated by CL and TL bands on the AuNPs-LFB. Negative results are indicated when only the CL band appears on the AuNPs-LFB. (**B**) Workflow of the HCV-RT-LAMP-AuNPs-LFB assay. The workflow comprises the following closely linked steps: rapid genomic RNA extraction (step 1), RT-LAMP amplification (step 2), and AuNPs-LFB visual interpretation (step 3). The entire detection process is complete within 40 min

In the current study, a novel reverse transcription loop-mediated isothermal amplification (RT-LAMP) approach was combined with the AuNPs-LFB platform, which could rapid, sensitive, specific, visual, and cost-saving identification of HCV by targeting the gene encoding the 5’untranslated region (5’UTR) [18]. The workflow and principle are illustrated in Fig. 1A and B. The feasibility of the assay was confirmed with clinical samples from patients with suspected HCV infections.

## Materials and methods

### Reagents

Crimson dye streptavidin-coated AuNPs (40 ± 5 nm, 10 mg/ml) were obtained from Bangs Laboratories Inc. (Fishers, IN, USA). Nitrocellulose (NC) membranes were purchased from Merck Millipore Co., Ltd. (Darmstadt, Germany). Rabbit anti-fluorescein antibody (anti-FAM; 0.2 mg/ml) and biotinylated bovine serum albumin (biotin-BSA; 4 mg/ml) were obtained from Abcam Co., Ltd. (Shanghai, China). The AuNPs-LFB containing sample pad, conjugate pad, NC membranes, and absorption pad were laminated on a plastic adhesive backing by HuiDeXin Biotech. Co., Ltd. (Tianjin, China) according to our design scheme (Fig. 1A). LAMP reaction kits containing colorimetric indicator (leuco-hydroxynaphthol blue, L-HNB) were purchased from HaiGene Biotech Co., Ltd. (Harbin, China), and stored at -20 ℃. Nucleic acid releasing agents were purchased from GenDx Biotech Co., Ltd. (Suzhou, China) (stored at -20 ℃). Commercial RT-qPCR diagnostic kits for HCV were purchased from Xi’an Tianlong Technology Co., Ltd. (Xi’an, China) (stored at -20 ℃).

### Clinical specimens and target gene preparation

Ninety-six clinical serum samples were collected from patients with suspected HCV infections treated at Second Affiliated Hospital, Guizhou University of Traditional Chinese Medicine from June 2022 to April 2023, and stored at -80 ℃ before use. The hospital’s Human Ethics Committee approved the protocols for the lawful and ethical collection and analyses (Approval No. KYW2022034). Nucleic acids were rapidly extracted from each sample using Nucleic Acid Releasing Agent (GenDx Biotech Co., Ltd.; Suzhou, China) according to the manufacturer’s instructions [19]. All of the extracted RNA samples were stored at -80 ℃ for subsequent analysis.

The full-length HCV 5’UTR gene sequences of five dominant subtypes in China (1b, 2a, 3b, 6a, and 3a) were obtained from the GenBank database (respective GenBank Accession No. EU781827.1, HQ639944.1, JQ065709.1, AY859526.1, and D17763.1) [20, 21]. Each gene sequence was synthesized and cloned into the pUC57 vector. The initial concentration of each plasmid was 1 × 10^8^ copies per ml. HCV subtype 1b plasmid was the positive control.

### Preparation of AuNPs-LFB

A schematic diagram of the AuNPs-LFB is shown in Fig. 1A. Briefly, the biosensor (60 × 4 mm) is mainly consisted of four components: sample pad, conjugate pad, NC membrane, and absorbent pad. Crimson dye streptavidin (SA)-AuNPs were gathered on the conjugate pad. Rabbit anti-FAM and Biotin-BSA were fixed onto the NC membrane of the test line (TL) and control line (CL), respectively. Each line was separated by 5 mm. Finally, the four components were banded together on a plastic card using adhesive backing. The AuNPs-LFB used in the current study was manufactured by Tian-Jin HuiDeXin Biotech Co., Ltd. (Tianjin, China) based on our design specifications and stored at room temperature before use, and exhibited excellent storage stability [22, 23].

### Primer design

The HCV 5’UTR gene was used as the target sequence in the assay. 5’UTR genes from the five dominant HCV subtypes in China (1b, 2a, 3b, 6a, and 3a) were aligned by DNASTAR software (DNASTAR Inc., Madison, WI, USA) (Supplementary Figure S1). The conserved sequences were used for LAMP primer design with Primer Explorer v.5 (http://primerexplorer.jp/e/) and Primer Premier v.5.0 software. The set of LAMP primers contained F3, B3, FIP, BIP, LF, and LB. For the AuNPs-LFB test, the 5’ ends of the FIP and LF primers were labeled with FAM and biotin, respectively. The primers and modifications were shown in Table 1. Location details were shown in Supplementary Figure S1. All primers were synthesized and purified by high-performance liquid chromatography by TsingKe Biotech Co., Ltd. (Beijing, China).

**Table 1:** The HCV-RT-LAMP-AuNPs-LFB primers used in this study

Primer name	Sequence and modifications	Length	Gene
F3	5’-TCGT(A/G)CAGCCTCCAGG(A/C)-3’	17 nt	5’UTR
B3 FIP	5’-GGTCTACGAGACCTCCCG-3’ 5’-AAGAAAGGACCC(A/G)GTC(G/A/T)(C/T)CC(C/T)-CATAGT(A/G)GTCTGCGGAACC-3’	18 nt 40 mer	
FIP*	5’-FAM-AAGAAAGGACCC(A/G)GTC(G/A/T)(C/T)CC(C/T)-CATAGT(A/G)GTCTGCGGAACC-3’	40 mer	
BIP LF LF* LB	5’-CCGC(A/G)AGA(C/T)(C/T)(A/G)CTAGCCGA-GCACCCTATCAGGCAGTACC-3’ 5’-C(A/G)ATTCCGGTGTACTCAC-3’ 5’-Biotin-C(A/G)ATTCCGGTGTACTCAC-3’ 5’-TAG(C/T)GTTGGGT(C/T)GCGAAAG-3’	39 mer 18 nt 18 nt 19 nt	

*Note* FIP*, 5’-labeled with FAM, LF*,5’-labeled with biotin, when used for the AuNPs-LFB assay

*Abbreviations* FAM, 6-carboxy-fluorescein; nt, nucleotide; mer, monomeric unit

### Standard HCV-RT-LAMP assay

The 25 µl LAMP reaction system consisted of 2.5 µl 10 × *Bst* 4.2 Buffer (Mg^2+^ free), 1 µl *Bst* 4.2 DNA/RNA polymerase (8 U), 1.5 µl 100 mM Mg^2+^, 3 µl of dNTP Mixture (10 mM), 0.1 µM F3 and B3, 0.4 µM FIP or FIP* (for AuNPs-LFB only) and BIP, 0.2 µM LF or LF* (for AuNPs-LFB only) and LB, 1.5 µl L-HNB (for colorimetry only), template (1 µl standard plasmid or 5 µl of each sample), and double-distilled water to bring the volume to 25 µl.

Four monitoring methods, agarose gel electrophoresis, real-time turbidity, visual detection (L-HNB), and AuNPs-LFB, were used for analyzed RT-LAMP amplicons. For agarose gel electrophoresis, the agarose gel presented ladder-like bands indicated a positive outcome, with no bands indicating a negative result. For L-HNB, a reaction mixture that turned from deep violet to light green indicated positive HCV-RT-LAMP amplification. A mixture that remained deep violet throughout the reaction indicated a negative outcome. For real-time turbidity assessment, a turbidity value of > 0.1 indicated a successful HCV-RT-LAMP result. For detection in the AuNPs-LFB platform, two visible crimson line (CL and TL) observed simultaneously on the biosensor indicated a positive HCV-RT-LAMP outcome. In negative and blank controls, only the CL line was appeared.

### Optimization of HCV-RT-LAMP-AuNPs-LFB assay conditions

HCV-RT-LAMP reaction temperatures ranging from 63 to 70℃ (in 1℃ increments) were tested to establish the optimal reaction temperature in the standard HCV-RT-LAMP reaction system. The products were analyzed by real-time turbidity (LA-500, Eiken Chemical Co., Ltd., Japan). Next, the optimal RT-LAMP amplification time was confirmed by using HCV-RT-LAMP reaction times of 10 to 40 min (in 10-min increments). The detection process was performed under the optimized reaction temperature, and the products were monitored simultaneously by L-HNB and AuNPs-LFB. Each test was tested three times at differently days.

### Sensitivity of HCV-RT-LAMP-AuNPs-LFB assay

The HCV 5’UTR-plasmid templates were 10-fold serially diluted (range from 2.0 × 10^3^ copies to 1 copy per test) to determine the assay’s limit of detection (LoD) using the established optimum reaction conditions. Results were detected simultaneously by L-HNB and AuNPs-LFB. Each test was repeated three times at differently days.

### Specificity and Selectivity of HCV-RT-LAMP-AuNPs-LFB assay

The specificity and selectivity of HCV-RT-LAMP-AuNPs-LFB assay were evaluated via five HCV 5’UTR-plasmid templates (HCV subtypes 1b, 2a, 3b, 6a, and 3a), six HCV RNAs (confirmed by RT-PCR), and other microbial nucleic acid templates such as Hepatitis B virus, Human immunodeficiency virus, Epstein-Barr virus, Coxsackie virus CAV16,, Human papillomavirus, Human enterovirus EV71, Influenza A virus, Influenza B virus, *Cryptococcus neoformans*, *Enterococcus faecium*, *Salmonella enteritidis*, *Shigella bogdii*, *Staphylococcus aureus*, *Streptococcus pyogenes*, *Streptococcus pneumoniae*, *Escherichia coli, Pseudomonas aeruginosa*, *Klebsiella pneumoniae*, *Mycobacterium tuberculosis*, *Brucella*, and their mixtures (Table 2). All the other microbial nucleic acid templates used in the current study were extracted through Virus DNA/RNA Extraction Kits or Bacterial Genomic DNA Extraction Kits (Xi’an Tianlong Technology Co., Ltd.; Xi’an, China), and the Concentration and purity were identified with a Nano Drop ND-2000 (Beijing, China) at A260/280. The DNA/RNA templates were stored at -80 °C before use. The detection process was performed under the optimized reaction conditions with the same set of HCV-LAMP primers. All of the amplicons were detected using AuNPs-LFB, with distilled water (DW) as the blank control (BC). Each test was performed in triplicate at differently days.

**Table 2:** Pathogens used in this study

No.	Pathogen	Source of pathogens^a^	No. of strains	HCV-LAMP-AuNPs LFB result^b^
1	HCV 1b 5’UTR-plasmid	Constructed by Tsingke Biotech (Beijing, China)	1	P
2	HCV 2a 5’UTR-plasmid	Constructed by Tsingke Biotech (Beijing, China)	1	P
3	HCV 3b 5’UTR-plasmid	Constructed by Tsingke Biotech (Beijing, China)	1	P
4	HCV 6a 5’UTR -plasmid	Constructed by Tsingke Biotech (Beijing, China)	1	P
5	HCV 3a 5’UTR -plasmid	Constructed by Tsingke Biotech (Beijing, China)	1	P
6	HCV clinical samples	2nd GZUTCM	6	P
7	Hepatitis B virus	2nd GZUTCM	1	N
8	Human immunodeficiency virus	GZCCL	1	N
9	Epstein-Barr virus	2nd GZUTCM	1	N
10	Coxsackie virus CAV16	GZCDC	1	N
11	Human papillomavirus	GZCCL	1	N
12	Human enterovirus EV71	GZCDC	1	N
13	Influenza A virus	GZCDC	1	N
14	Influenza B virus	GZCDC	1	N
15	*Cryptococcus neoformans*	2nd GZUTCM	1	N
16	*Enterococcus faecium*	2nd GZUTCM	1	N
17	*Salmonella enteritidis*	2nd GZUTCM	1	N
18	*Shigella bogdii*	2nd GZUTCM	1	N
19	*Staphylococcus aureus*	ATCC25923	1	N
20	*Streptococcus pyogenes*	2nd GZUTCM	1	N
21	*Streptococcus pneumoniae*	2nd GZUTCM	1	N
22	*Escherichia coli*	ATCC8739	1	N
23	*Pseudomonas aeruginosa*	ATCC27853	1	N
24	*Klebsiella pneumoniae*	ATCC700603	1	N
25	*Mycobacterium tuberculosis*	GZCDC	1	N
26	*Brucella*	GZCDC	1	N

*Note *^a^ATCC, American Type Culture Collection; 2nd GZUTCM, the Second Affiliated Hospital, Guizhou University of Traditional Chinese Medicine; GZCCL, Guizhou Provincial Center for Clinical Laboratory; GZCDC, Guizhou Provincial Center for Disease Control and Prevention

^b^P, Positive; N, Negative

### Feasibility of HCV-RT-LAMP-AuNPs-LFB for clinical samples

The feasibility of the assay for clinical applications was assessed. Ninety-six serum specimens were collected from patients with suspected HCV infections. The genomic RNA was rapidly isolated using Nucleic Acid Releasing Agents (GenDx Biotech Co., Ltd.) as previously described [19]. All the samples were simultaneously detected by the HCV-RT-LAMP-AuNPs-LFB and HCV-RT-qPCR assays. The HCV-RT-LAMP-AuNPs-LFB assay was performed as previously described. HCV-RT-qPCR was performed using HCV Nucleic Acid Assay Kits (Xi’an Tianlong Technology Co., Ltd; Xi’an, China) on an Applied Biosystems™ 7500 Real-Time PCR System (Life Technologies; Singapore). Sample copy number was determined using a standard curve. HCV concentrations > 50 IU/ml (approximately 45 copies/ml) were deemed a positive result according to the manufacturer’s recommendations. HCV-positive samples were further identified with Sanger sequencing (Dian Medical Laboratory Center Co., Ltd.; Hangzhou, China). The detection was carried out at biosafety level 2 (BSL-2) based on the WHO Biosafety Manual (3rd Edition). The outcomes of the HCV-RT-LAMP-AuNPs-LFB assay were compared with RT-qPCR results. The statistical parameters were calculated through online tool from MedCalc (http://www.medcalc.org/calc/diagnostic_test.php) [24].

## Results

### HCV-RT-LAMP-AuNPs-LFB assay system overview

The principle and workflow of HCV-RT-LAMP-AuNPs-LFB assay are presented in Fig. 1A and B. Briefly, genomic RNA was rapidly isolated within 5 min (Fig. 1B, step 1). The target gene was amplified with RT-LAMP for 30 min at a constant temperature of 67 °C. In the HCV-RT-LAMP reaction system, FAM and biotin were attached to the HCV 5′-UTR unique LAMP primer set. HCV-FIP* and LF* were labeled at the 5′ end with FAM and biotin, respectively. With the *Bst* 4.2 DNA/RNA polymerase activation, the HCV-RT-LAMP products were simultaneously labeled with FAM and biotin. The labeled LAMP products were analyzed visually with AuNPs-LFB platform. (Fig. 1B, step 2). The results were analyzed visually using AuNPs-LFB within 2 min (Fig. 1B, step 3). The whole detection process was complete within 40 min.

The AuNPs-based LFB divided into four components: a sample pad, a conjugate pad, a detection region (nitrocellulose membrane), and an absorbent pad. Crimson red dye streptavidin-coated AuNPs were deposited at the conjugate pad. Anti-FAM antibody and biotinylated bovine serum albumin (biotin-BSA) were immobilized at the detection region (nitrocellulose membrane, NC) of test line (TL) and control line (CL), respectively. the four components were assembled and packaged in a plastic cassette.

For AuNPs-LFB identification, HCV-RT-LAMP products (2.0 µl) and running buffer (100 µl) were added to an AuNPs-LFB sample pad (Fig. 1A, I). The running buffer containing the HCV-RT-LAMP products moved long the AuNPs-LFB by capillary action, and the crimson dye SA-AuNPs were rehydrated and the FAM/biotin-labeled HCV-RT-LAMP products were integrated with streptavidin-AuNPs (Fig. 1A, II). For positive results, the FAM/biotin-labeled HCV-RT-LAMP products were bound by anti-FAM at the TL, and the SA-AuNPs were fixed by biotin-BSA at the CL (Fig. 1A, III). The interpretation of the HCV-RT-LAMP-AuNPs-LFB results is outlined in Fig. 1A (IV). In a positive outcome, both CL and TL appeared simultaneously on the AuNPs-LFB. For negative results, only CL was presented on the biosensor.

### Demonstration and detection of HCV-RT-LAMP products

**Fig. 2:**
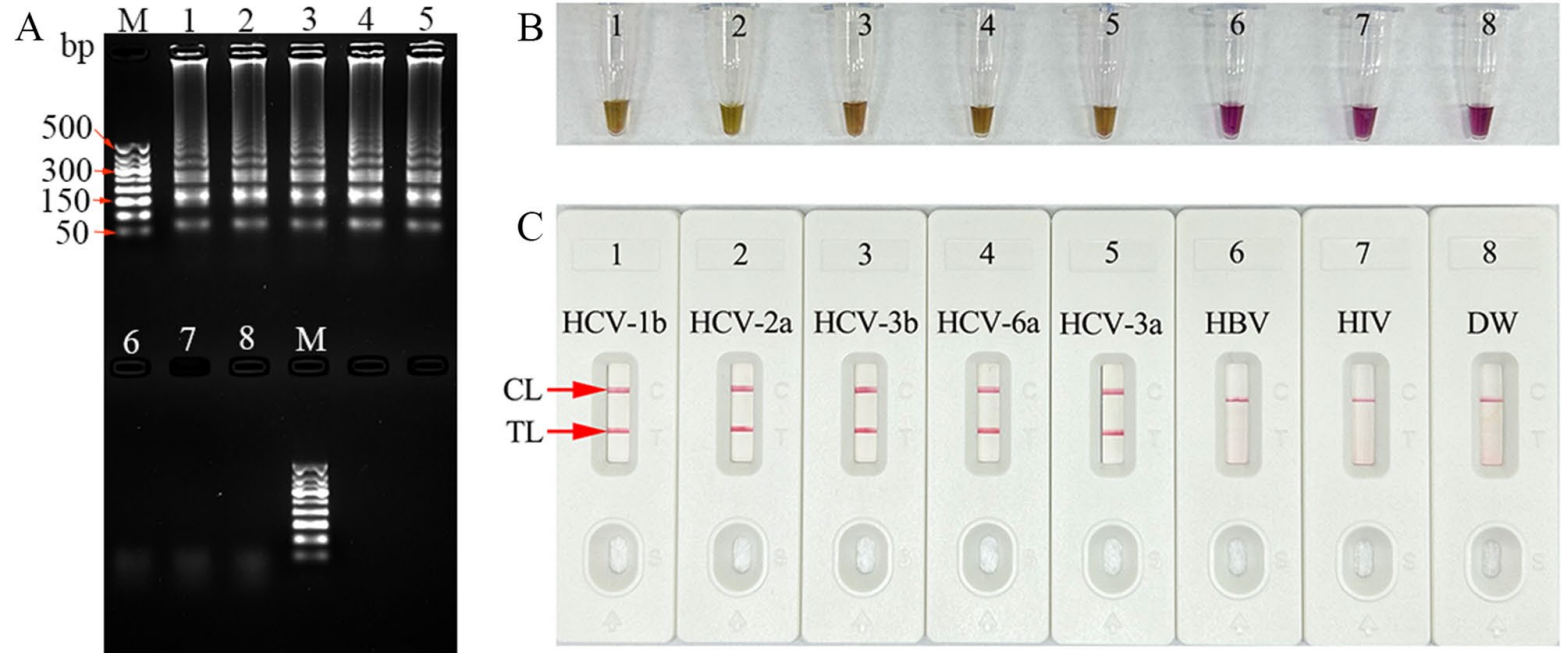
Verification of HCV-RT-LAMP products. The HCV-RT-LAMP products were identified simultaneously using (**A**) 2% agarose gel electrophoresis, (**B**) color change (L-HNB), and (**C**) AuNPs-LFB. Templates of 1–8 were HCV-1b plasmid, HCV-2a plasmid, HCV 3b plasmid, HCV-6a plasmid, HCV-3a plasmid, hepatitis B virus (HBV), human immunodeficiency virus (HIV), and distilled water (DW), respectively. CL: control line; TL: test line

The effectiveness of the HCV RT-LAMP primers was assessed. Five synthetic 5’UTR-plasmid DNA templates (HCV subtypes 1b, 2a, 3b, 6a, and 3a) were used for HCV RT-LAMP reaction for 1 h at a constant temperature of 65 °C. The products were identified through 2% agarose gel electrophoresis (Fig. 2A), colorimetric indicator (L-HNB) (Fig. 2B), and AuNPs-LFB (Fig. 2C). The templates from five synthetic 5’UTR plasmids were significantly amplified, while no amplification was observed from the templates of hepatitis B virus (HBV), HIV, and distilled water (DW) controls. These data suggested that the LAMP primer set targeting the 5’UTR gene was an appropriate candidate for the development of the HCV-RT-LAMP-AuNPs-LFB assay.

### Optimal amplification temperature for HCV-RT-LAMP assay

**Fig. 3:**
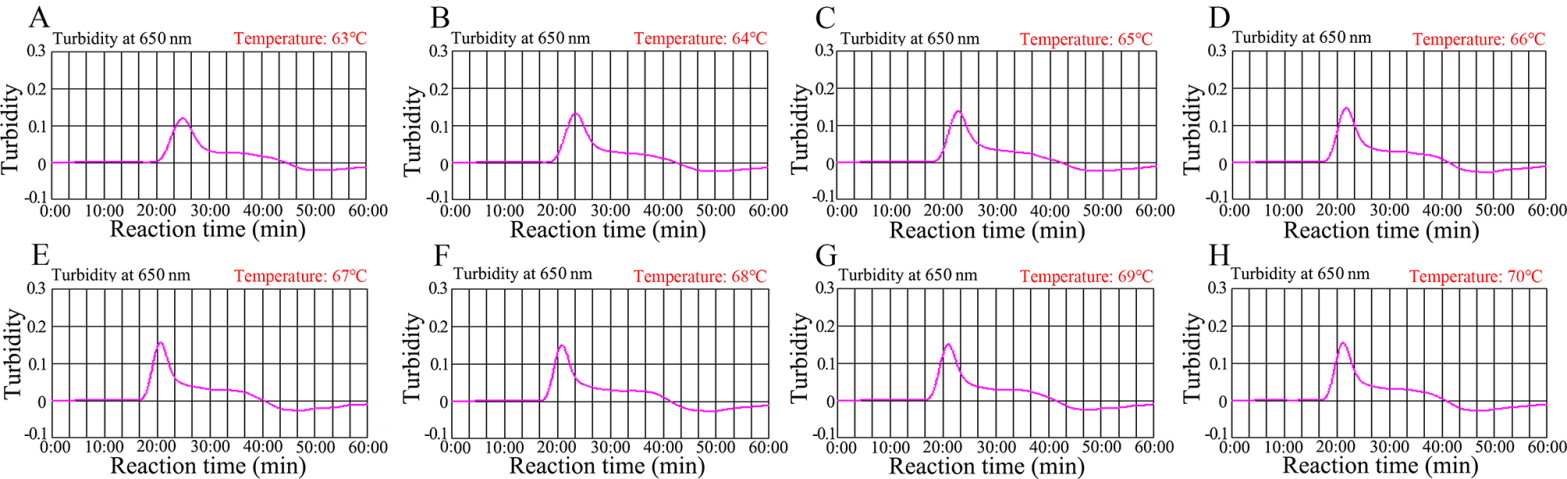
Temperature optimization for HCV-RT-LAMP amplification. The RT-LAMP amplifications for detection of HCV were monitored using real-time turbidity (LA-500, Eiken Chemical Co., Ltd., Japan). The corresponding curves of amplicons are displayed in the graphs. Turbidity > 0.1 indicated a positive value. Eight kinetic graphs were obtained at different temperatures (63–70 °C, 1 °C intervals) with 2 × 10^3^ copies of the target gene (**A-H**). The graphs from **E** (67 °C) showed robust amplification

Reaction temperature is important for high-efficiency LAMP amplification. The amplification temperature for LAMP reaction was optimized using temperatures ranging from 63 to 70℃ with 5’UTR plasmids (copy number of 2.0 × 10^3^). The outcomes of HCV-RT-LAMP amplification were tracked with real-time turbidity measurements, and the kinetic graph was generated from each reaction temperature (Fig. 3A-H). The results indicated that the robust amplification of HCV-RT-LAMP occurred at 67℃ (Fig. 3E).

### Sensitivity of HCV-RT-LAMP-AuNPs-LFB assay

**Fig. 4:**
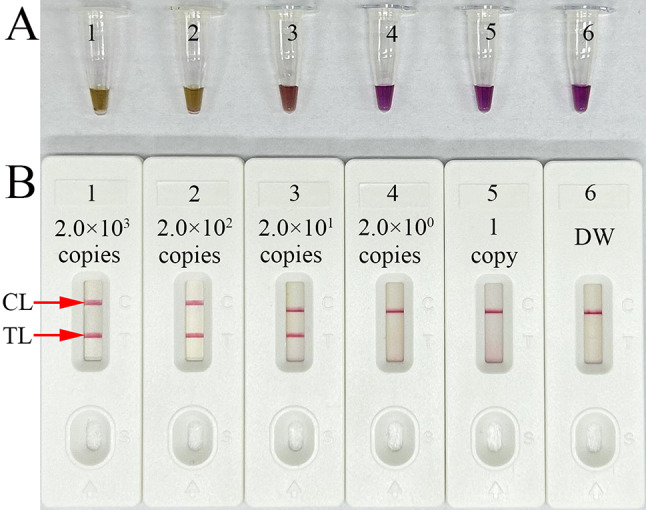
Sensitivity analysis of the HCV-RT-LAMP-AuNPs-LFB assay with serial dilutions of nucleic acid template. Visual reagent (L-HNB) and AuNPs-LFB approaches were simultaneously used to readout the HCV-RT-LAMP outcomes. L-HNB (**A**)/AuNPs-LFB (**B**); 1–6 represent the HCV plasmid concentrations of 2.0 × 10^3^ copies, 2.0 × 10^2^ copies, 2.0 × 10^1^ copies, 2.0 × 10^0^ copies, and 1 copy per test and distilled water (DW), respectively. The limit of detection (LoD) of the HCV-RT-LAMP assay was 20 copies per test. CL: control line; TL: test line

Serial dilutions of nucleic acid templates (5’UTR plasmids) from 2.0 × 10^3^ copies to 1 copy was used to determine the LoD. HCV-RT-LAMP reactions were performed for 1 h 67℃, and the outcomes were analyzed using colorimetric indicator (L-HNB) and AuNPs-LFB. The assay sensitivity was 20 copies per test and the visual AuNPs-LFB readouts were consistent with L-HNB detections (Fig. 4A and B).

### Optimal reaction time for HCV-RT-LAMP-AuNPs-LFB assay

**Fig. 5:**
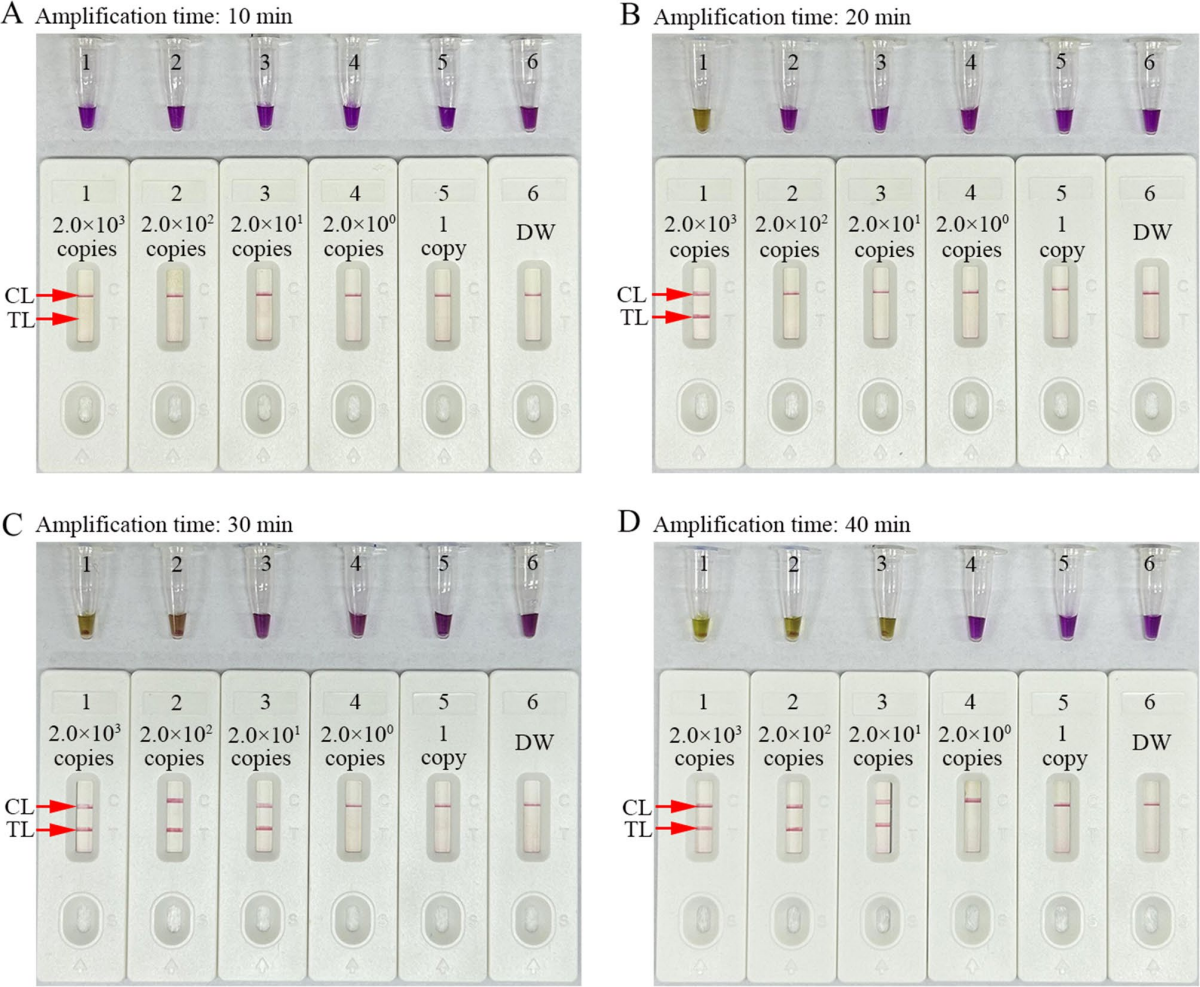
Amplification time optimization for the HCV-RT-LAMP-AuNPs-LFB assay. Four RT-LAMP reaction times, including 10 min (**A**), 20 min (**B**), 30 min (**C**), and 40 min (**D**), were evaluated at optimal reaction conditions. Tube/biosensor 1–6 represent HCV plasmid concentrations of 2.0 × 10^3^ copies, 2.0 × 10^2^ copies, 2.0 × 10^1^ copies, 2.0 × 10^0^ copies, and 1 copy per test and distilled water (DW), respectively. The results were analyzed simultaneously using visual reagent L-HNB and AuNPs-LFB. The signal of the LoD appeared with a 30 min reaction time through AuNPs-LFB. CL, control line; TL, test line

Multiple times (10, 20, 30 and 40 min) were tested to confirm the optimal reaction time for the stage of the RT-LAMP amplification at the optimal amplification temperature of 67℃ (Fig. 5). The outcomes of RT-LAMP amplification were tracked with colorimetric indicator (L-HNB) and AuNP-LFB platform. The LoD of 20 copies of the HCV-5’UTR-plasmids were detected when the amplification time was 30 min through AuNPs-LFB (Fig. 5C). This reaction time was subsequently used for HCV-RT-LAMP-AuNPs-LFB assay procedures. However, the LoD was tested require 40 min using L-HNB (Fig. 5D). The assay included rapid nucleic acid extraction (5 min), RT-LAMP reaction (30 min), and AuNPs-LFB readout (within 2 min), and was completed within 40 min.

### Specificity and Selectivity of HCV-RT-LAMP-AuNPs-LFB assay

**Fig. 6:**
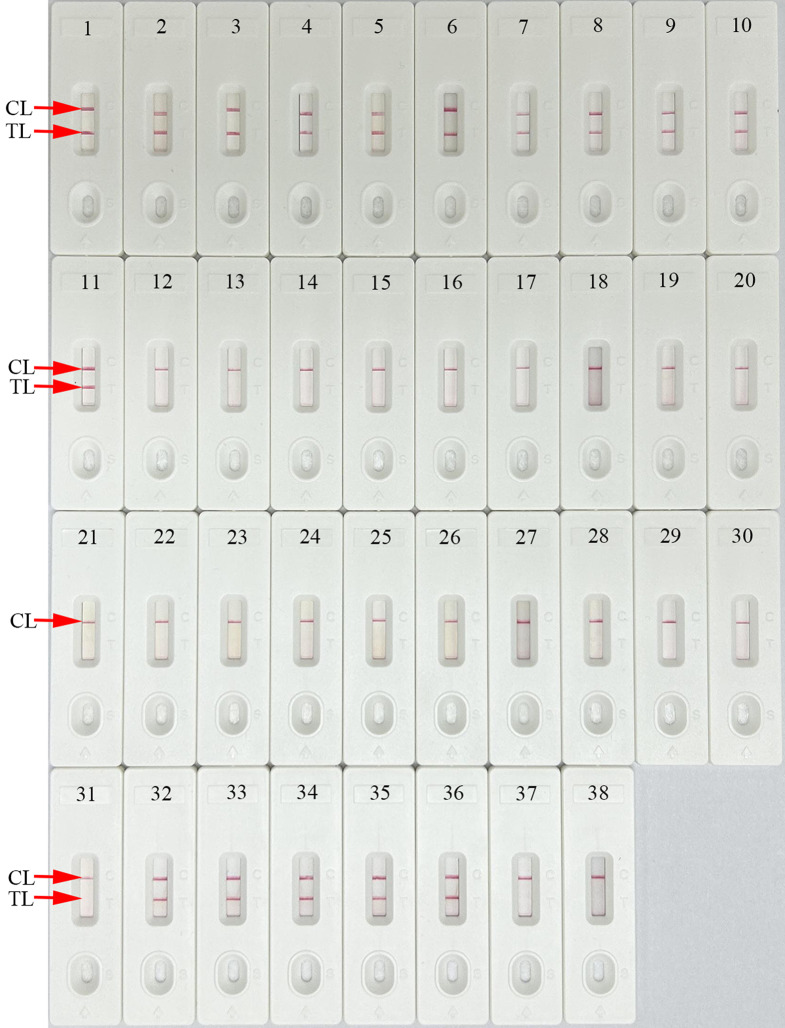
HCV-RT-LAMP-AuNPs-LFB assay specificity with different strains. Assay specificity was evaluated using different nucleic acid templates. Each result was tested through AuNPs-LFB: 1–5, HCV 1b, 2a, 3b, 6a, and 3a 5’UTR plasmids; 6–11, HCV clinical samples; 12, Hepatitis B virus; 13, Human immunodeficiency virus; 14, Epstein-Barr virus; 15, Coxsackie virus CAV16; 16, Human papillomavirus; 17, Human enterovirus EV71; 18, Influenza A virus; 19, Influenza B virus; 20, *Cryptococcus neoformans*; 21, *Enterococcus faecium*; 22, *Salmonella enteritidis*; 23, *Shigella bogdii*; 24, *Staphylococcus aureus*; 25, *Streptococcus pyogenes*; 26, *Streptococcus pneumoniae*; 27, *Escherichia coli*; *28, Pseudomonas aeruginosa*; 29, *Klebsiella pneumoniae*; 30, *Mycobacterium tuberculosis*; 31, *Brucella*; 32, Other microbial nucleic acid mixtures containing HCV-1b plasmids; 33, Other microbial nucleic acid mixtures containing HCV-2a plasmids; 34, Other microbial nucleic acid mixtures containing HCV-3b plasmids; 35, Other microbial nucleic acid mixtures containing HCV-6a plasmids; 36, Other microbial nucleic acid mixtures containing HCV-3a plasmids; 37, Other microbial nucleic acid mixtures; 38, distilled water (DW). CL, control line; TL, test line

The specificity and selectivity of HCV-RT-LAMP-AuNPs-LFB assay was verified by testing the 5’UTR plasmids of the five HCV subtypes (1b, 2a, 3b, 6a, and 3a) prevalent in China, positive HCV clinical samples (confirmed by RT-qPCR), other 20 microbes, and their mixtures (Table 2). The HCV-RT-LAMP-AuNPs-LFB assay was performed under optimal reaction condition, and the results were readout with AuNPs-LFB platform. Positive results were observed only with nucleic acid extracted from HCV samples and the mixtures containing HCV nucleic acid. Non-HCV microbes and blank control showed negative outcomes, and no cross-reactions were observed from the HCV-RT-LAMP-AuNPs-LFB assay (Table 2; Fig. 6). The findings indicated that the HCV-RT-LAMP-AuNPs-LFB assay exhibited high specificity and anti-interference. In addition, each test of this study was repeated three times at differently days, and showed good reproducibility.

### Clinical feasibility for HCV-RT-LAMP-AuNPs-LFB assay

To further evaluate the HCV-RT-LAMP-AuNPs-LFB platform as a valuable tool for HCV identification, ninety-six serum samples from patients with suspected HCV infection were tested simultaneously using the HCV-RT-LAMP-AuNPs-LFB assay and HCV-RT-qPCR. The HCV-RT-LAMP-AuNPs-LFB assay verified 46 serum samples as HCV-positive. All were also HCV-positive using RT-qPCR. To verify their subtypes, all HCV-RT-qPCR positive samples were analyzed by Sanger sequencing at Dian Medical Laboratory Center Co., Ltd. (Hangzhou, China). The results showed that twenty-four samples were recognized as subtype 1b, fourteen samples as subtype 2a, three as subtype 3b, three as subtype 6a, two as subtype 3a (Table 3 and Supplementary TableS1). Compared with the HCV-RT-qPCR technology, the HCV-RT-LAMP-AuNPs-LFB sensitivity and specificity were 100.00% (95% CI: 92.29–100.00%) and 100% (95% CI: 92.89–100.00%), respectively (Table 3).

**Table 3:** Comparing HCV levels in clinical samples using RT-qPCR and our HCV-RT-LAMP-AuNPs-LFB methods

HCV-RT-LAMP-AuNPs-LFB	HCV-RT-qRCR (reference method)			Sensitivity		Specificity	
	Positive	Negative	Total	Value	95% CI	Value	95% CI
Positive	46 (Subtype 1b, 24; subtype 2a, 14; subtype 3b, 3; subtype 6a, 3; subtype 3a, 2)	0	46	100.00%	92.92%-100.00%	100%	92.89%- 100.00%
Negative	0	50	50				
Total	46	50	96				

## Discussion

Here, a novel HCV-RT-LAMP-AuNPs-LFB diagnosis platform, which integrated AuNPs-LFB identification with RT-LAMP amplification, was successfully developed for the highly specific, rapid, cost-saving, and visual identification of HCV. The feasibility of our platform was confirmed through clinical sera from individuals with suspected HCV infection, and the results were compared with a commercial HCV-RT-qPCR assay.

HCV is one of the major human viruses that cause of chronic liver disease [5]. HCV infections are often asymptomatic. If the infection is not treated in a timely manner, diseases including hepatic decompensation, hepatocirrhosis, and hepatocellular carcinoma often develop [1–3]. A sensitive, rapid, easy-to-use, and cost-saving diagnosis platform that can provide early viral detection is critical for prescribing DAAs and preventing HCV transmission. Conventionally, serological tests were first developed for blood donors screening and prevent blood transmission. Anti-HCV antibodies (Ab) are generally identified using enzyme linked immunosorbent assay (ELISA). Nevertheless, HCV-Ab can be detected within 4–8 weeks after infection [7, 8]. Moreover, immunosuppressed infected individuals can have false-negative outcomes. In recent decades, reverse transcription polymerase chain reaction technology was used to identify HCV infection, which has good sensitivity and can detect viremia in acute infection during the window period (when HCV-Ab assay is still negative) [25]. However, this assay is inappropriate in low- and middle-income countries owing to the high costs of thermo-cycling instruments and need for trained technicians. The developed HCV-RT-LAMP-AuNPs-LFB diagnosis platform can be done isothermally using basic instruments, such as a metal bath, water bath, or even a thermos cup. The results can be visually readout with the AuNPs-LFB system. In our assay, crude nucleic acid can be used for RT-LAMP amplification by *Bst* 4.2 DNA/RNA polymerase, which has fewer inhibitors than the *Taq* DNA polymerase used in traditional PCR techniques [26]. Hence, the HCV-RT-LAMP-AuNPs-LFB detection procedure can be completed within 40 min, including nucleic acid extraction (5 min), RT-LAMP amplification (30 min), and AuNPs-LFB visual interpretation (within 2 min).

In the current study, the HCV 5’UTR target gene was amplified through RT-LAMP. This newly developed gene amplification method can robustly amplify target sequences at a fixed temperature [10, 27, 28]. The specific amplicons were generated in the RT-LAMP reaction system by *Bst* 4.2 DNA/RNA polymerase and six specific primers that span eight unique segments of the target sequence. The set of HCV RT-LAMP primers included two outer primers (F3 and B3), two inner primers (FIP and BIP), and two loop primers (LF and LB). Here, we successfully devised a set of specific primers based on the 5’UTR gene from the five prevalent HCV subtypes (1b, 2a, 3b, 6a, and 3a) in China [20, 21]. RT-LAMP pre-amplification was then optimized, with a temperature of 67 °C and reaction time of 30 min. The specificity of the HCV-RT-LAMP-AuNPs-LFB platform was verified with HCV strains and other microbes. As expected, the HCV-RT-LAMP-AuNPs-LFB detection system was extremely specific for the identification of HCV strains. No cross-reactions with other pathogens occurred. The sensitivity of our assay was assessed by diluting the synthesized 5’UTR plasmids from 2.0 × 10^3^ copies to 1 copy prior to testing. The results confirmed that the novel assay can detect as few as 20 copies. More importantly, we also successfully applied the HCV-RT-LAMP-AuNPs-LFB assay to test clinical samples. The results confirmed that our assay can effectively identify HCV in clinical samples. Testing of clinical samples with significantly lower viral loads using our assay is planned and will more comprehensively determine its clinical applications.

In previous studies, RT-LAMP was used for the identification of HCV. Hongjaisee et al. (2021) combined RT-LAMP with naked-eye visual color detection of HCV [29]. However, the results were ambiguous when the HCV-RT-LAMP product concentrations were low. Kham-Kjing, et al. (2022) combined RT-LAMP with the clustered regularly interspaced short palindromic repeats-CRISPR-associated protein 12a (CRISPR–Cas12a) for testing HCV [30], this assay is high sensitivity and specificity. However, the HCV-RT-LAMP products must be identified with Cas12a-crRNA complex, and then the results were readout through lateral flow strips or a fluorescence detector, which is complicated operation and time-consuming (more than 1 h). In this study, for the first time, we combined LAMP amplification with gold nanoparticle-based lateral flow biosensor (AuNPs-LFB) for visual identification of HCV, which is more convenience and rapid than that of methods. AuNPs-LFB is a label-based biosensor rely on the specific properties of labels for detecting a particular target, which is promising for POC testing due to its speed, high sensitivity, good selectivity, cost-saving, visual readout, and success using low sample volumes [31]. In the current study, the AuNPs-LFB can visually interpret HCV-RT-LAMP outcomes with anti-FAM and BSA-biotin anchored on NC membranes. For positive outcomes, the FAM/biotin-labeled HCV-RT-LAMP products were captured at the TL and SA-AuNPs complexes were captured by biotin-BSA and visually readout at the CL. For negative outcomes, only the SA-AuNPs were arrested by biotin-BSA at the CL. In the current study, agarose gel electrophoresis, real-time turbidimetry, and visual reagent (L-HNB) were also used to interpret HCV-RT-LAMP results. The first two approaches require expensive devices and may not be available in many resource-limited regions. The L-HNB method is visual and equipment-free, but need more amplification time for detection of the LoD, and the visual result was ambiguous when the amplicons were low (Fig. 5D). The AuNPs-LFB is easy to operate and inexpensive (~ US$2.0 per test). Therefore, the total cost of each test, including the genomic RNA isolation (~ US$0.5), RT-LAMP reactions (~ US$1.5), and AuNPs-LFB readout (~ US$2.0), is approximately US$4.0. We summarized the commonly used methods for HCV detection [32–35], their analytical properties and advantages/disadvantages were shown in Table 4. In addition, label-free biosensors, such as field-effect transistor-based biosensors, magnetoelastic biosensors, and optical-based biosensors, are another increasing awareness of novel biomolecule detection techniques that do not require labeling of ligand or the receptor, which can screen for biologically active molecular interactions and cellular responses, they are suitable for the target molecules that are not labeled or the screening of analytes which are not easy to tag [36, 37].Our HCV-RT-LAMP-AuNPs-LFB assay does have some weakness. First, HCV has high genetic heterogeneity and various subtypes can be present. Our RT-LAMP primers were selected to specifically identify the five dominant subtypes in China (1b, 2a, 3b, 6a, and 3a). Further refinement using more diverse RT-LAMP primers will be necessary to identify many more HCV subtypes. Second, the HCV-RT-LAMP amplification tube must be opened for AuNPs-LFB identification, increasing the risk of carry-over contamination. To avoid nucleic acid contamination, nucleic acid contamination scavenger must be applied by spraying soon after completing each AuNPs-LFB identification. To better adapt the platform for clinical applications, it will be necessary to refine the HCV-RT-LAMP-AuNPs-LFB assay system and devise a convenient apparatus that avoids tube opening.

**Table 4:** Comparison of the commonly used methods for HCV detection

Technology	Limit of detection	Sensitivity (%)	Specificity (%)	Advantages	Disadvantages	Reference
Enzyme linked immunosorbent assay	-	98.7	100	High specificity and easy operation	Cannot detect viremia in acute infection during the window period	[32]
Real-time PCR assay	100 IU/ml	99.0	100.0	Quantitative analysis	Require expensive equipment and trained technicians	[33]
RT-LAMP combined with visual reagent assay	10–100 ng/reaction	95.5	100	High specificity; without opening the reaction tubes for results identification	The results are ambiguous when the LAMP product concentrations are low	[29] Current study
RT-LAMP-coupled CRISPR-Cas12 assay	10 ng/µL	96	100	High sensitivity and specificity	The operation is complicated	[30]
RT-LAMP-AuNPs-LFB assay	20 copies/test	100 95% CI (92.92–100%)	100 95% CI (92.89–100%)	High sensitivity and specificity; accurate and visual interpretation; easy to operate and cost-saving	Need open the reaction tube for results identification	Current study
Bioelectric recognition assay	0.1 nmol/L	90.0	84.4	Aptamer/DNAzyme can replicate hundreds of times in a short time	Extremely complicated	[34]
Amperometric biosensor assay	1.82 × 10^− 21^ mol/L	-	-	High selectivity for substrates	Used biosensors exhibit limited stability	[35]

## Conclusions

In the current study, for the first time, we reported a novel HCV molecular diagnostic approach termed RT-LAMP isothermal amplification integrated with a visual AuNPs-LFB for the specific, sensitive, visual, and rapid detection of HCV in clinical settings. The HCV-RT-LAMP-AuNPs-LFB assay had an LoD of 20-copies, and exhibited high specificity and anti-interference. The entire identification procedure, including nucleic acid extraction (5 min), RT-LAMP amplification (30 min), and AuNPs-LFB visual interpretation (within 2 min), was completed within 40 min without the need for sophisticated equipment. Therefore, our assay meets the requirement of the World Health Organization ASSURED criteria for POC testing: affordable, sensitive, specific, user-friendly, rapid and robust, equipment-free, and deliverable to end users.

### Electronic supplementary material

Below is the link to the electronic supplementary material.

Supplementary Material 1

## Data Availability

The original contributions presented in the study are included in the article/supplementary material, further inquiries can be directed to the corresponding author.
