# Supplementary material for: Rapid, visual, label-based biosensor platform for identification of hepatitis C virus in clinical applications
Source: BMC Microbiol. 2024 Feb 28;24:68. doi: 10.1186/s12866-024-03220-9 (PMC10900634; doi:10.1186/s12866-024-03220-9)
Supplement: Supplementary file 1 — Supplementary Material 1 [file 12866_2024_3220_MOESM1_ESM.docx]

**Supplementary Materials**

**Rapid, Visual, Label-Based Biosensor Platform for Identification of Hepatitis C Virus in Clinical Applications**

Yuanfang Shi^1,2Δ^, Qingxue Zhou^3Δ^, Shilei Dong^4^, Qi Zhao^5^, Xue Wu^6^, Peng Yang^7^, Xiaoyan Zeng^2^, Xinggui Yang^8^, Yan Tan^9^, Xinhua Luo^10^, Zhenghua Xiao^1,5^, and Xu Chen^1,2,6*^

^1^The Second Clinical College, Guizhou University of Traditional Chinese Medicine, Guiyang, Guizhou, 550003, People’s Republic of China

^2^Central Laboratory of the Second Affiliated Hospital, Guizhou University of Traditional Chinese Medicine, Guiyang, Guizhou, 550003, People’s Republic of China

^3^Clinical Laboratory, Hangzhou Women’s Hospital, Hangzhou, Zhejiang 310008, People’s Republic of China

^4^Department of Clinical Laboratory, Zhejiang Hospital, Hangzhou, Zhejiang 310013, People’s Republic of China

^5^Department of gastroenterology, the Second Affiliated Hospital, Guizhou University of Traditional Chinese Medicine, Guiyang, Guizhou, 550003, People’s Republic of China

^6^Department of Scientific Research, the Second Affiliated Hospital, Guizhou University of Traditional Chinese Medicine, Guiyang, Guizhou, 550003, People’s Republic of China

^7^Clinical Laboratory, the Second Affiliated Hospital, Guizhou University of Traditional Chinese Medicine, Guiyang, Guizhou, 550003, People’s Republic of China

^8^Experiment Center, Guizhou Provincial Centre for Disease Control and Prevention, Guiyang, Guizhou, 550004, People’s Republic of China

^9^Clinical Laboratory, Guizhou Provincial Center for Clinical Laboratory, Guiyang, Guizhou, 550002, People’s Republic of China

^10^Department of Infectious Disease, Guizhou Provincial People’s Hospital, Guiyang, Guizhou, 550002, People’s Republic of China

^Δ^Drs. Yuanfang Shi and Qingxue Zhou have contributed equally to this work.

^*^Corresponding author:

Xu Chen, E-mail: [xuchen1220@126.com](mailto:xuchen1220@126.com)

Zhenghua Xiao, E-mail: xiaozhenghua097@126.com

**
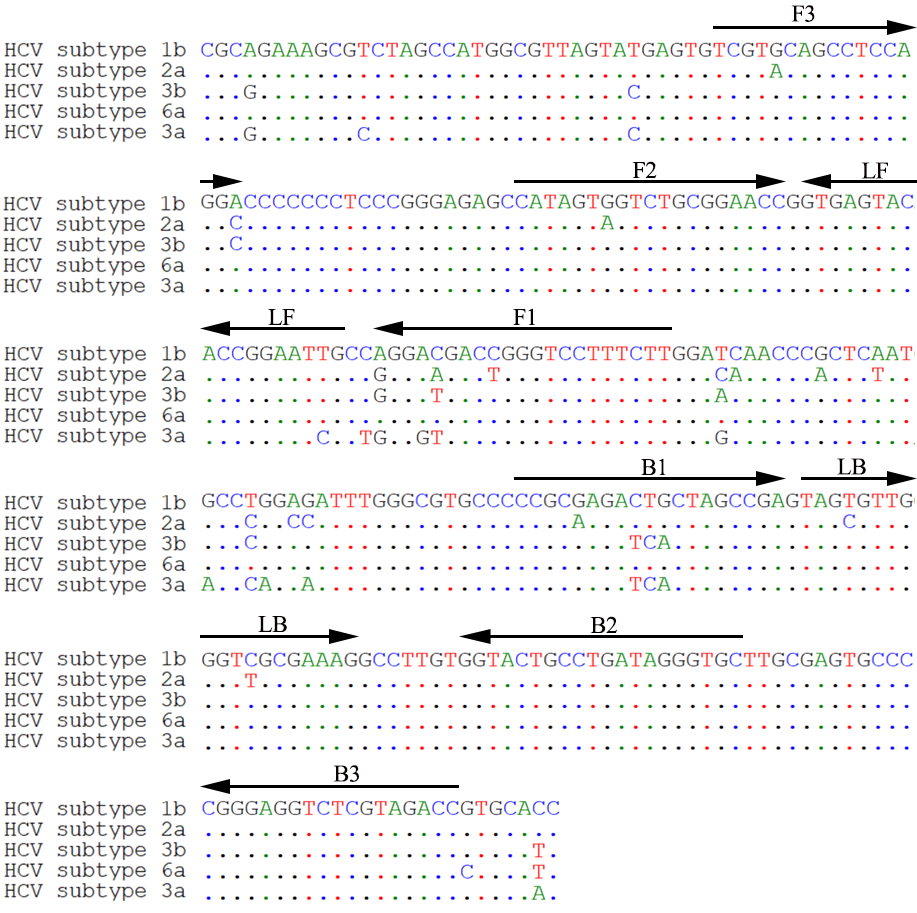
**

**Figure S1** Nucleotide sequences and locations of the 5’UTR genes from representative HCV subtypes (1b, 2a, 3b, 6a, and 3a) used to design the HCV-RT-LAMP primers. Right arrows and left arrows indicated the sense and complementary sequences which were used in this study, respectively.

**Table S1** Comparison of RT-qPCR and HCV-RT-LAMP-AuNPs-LFB assays for detection of HCV using clinical samples

| **Sample NO.** | **RT-qPCR result（copies）** | **HCV-RT-LAMP-AuNPs-LFB** | **Genotype** |
| --- | --- | --- | --- |
| Test 1 | 4.30×10^5^ | + | 1b |
| Test 2 | 1.08×10^7^ | + | 1b |
| Test 3 | 1.01×10^6^ | + | 1b |
| Test 4 | 1.46×10^7^ | + | 6a |
| Test 5 | 1.43×10^7^ | + | 1b |
| Test 6 | 3.20×10^6^ | + | 1b |
| Test 7 | 7.76×10^5^ | + | 1b |
| Test 8 | 3.91×10^6^ | + | 1b |
| Test 9 | 3.84×10^6^ | + | 2a |
| Test 10 | 3.95×10^6^ | + | 3b |
| Test 11 | 1.66×10^6^ | + | 1b |
| Test 12 | 1.27×10^6^ | + | 1b |
| Test 13 | 1.75×10^5^ | + | 2a |
| Test 14 | 6.56×10^4^ | + | 6a |
| Test 15 | 4.94×10^7^ | + | 3a |
| Test 16 | 1.81×10^7^ | + | 1b |
| Test 17 | 1.52×10^7^ | + | 1b |
| Test 18 | 5.42×10^3^ | + | 3a |
| Test 19 | 3.43×10^4^ | + | 1b |
| Test 20 | 3.62×10^5^ | + | 1b |
| Test 21 | 4.13×10^6^ | + | 2a |
| Test 22 | 8.70×10^5^ | + | 2a |
| Test 23 | 5.65×10^5^ | + | 1b |
| Test 24 | 1.32×10^6^ | + | 1b |
| Test 25 | 2.11×10^7^ | + | 2a |
| Test 26 | 1.16×10^6^ | + | 2a |
| Test 27 | 7.54×10^4^ | + | 1b |
| Test 28 | 2.39×10^5^ | + | 2a |
| Test 29 | 2.18×10^6^ | + | 1b |
| Test 30 | 4.34×10^3^ | + | 2a |
| Test 31 | 7.89×10^6^ | + | 3b |
| Test 32 | 5.58×10^4^ | + | 1b |
| Test 33 | 6.16×10^6^ | + | 1b |
| Test 34 | 2.52×10^6^ | + | 2a |
| Test 35 | 1.12×10^7^ | + | 6a |
| Test 36 | 1.09×10^6^ | + | 2a |
| Test 37 | 9.17×10^5^ | + | 1b |
| Test 38 | 4.42×10^5^ | + | 1b |
| Test 39 | 4.20×10^6^ | + | 2a |
| Test 40 | 7.58×10^4^ | + | 1b |
| Test 41 | 1.97×10^7^ | + | 2a |
| Test 42 | 7.73×10^6^ | + | 3b |
| Test 43 | 3.96×10^4^ | + | 2a |
| Test 44 | 4.62×10^6^ | + | 1b |
| Test 45 | 2.66×10^7^ | + | 1b |
| Test 46 | 2.98×10^6^ | + | 2a |
| Test 47-96 | — | — | — |

**Notice:** The RT-qPCR diagnosis was carried out using commercial real-time TaqMan PCR Kit (Xi’an Tianlong Technology Co., Ltd.; Xi’an China). The concentrations of HCV more than 45 copies will be considered as a positive result according to the manufacturer’s instructions. The HCV-RT-qPCR positive samples were analyzed with Sanger sequencing (Dian Medical Laboratory Center Co., Ltd.; Hangzhou, China).

+, Positive; —, Negative
